# Granular effects in sea ice rheology in the marginal ice zone

**DOI:** 10.1098/rsta.2021.0260

**Published:** 2022-10-31

**Authors:** A. Herman

**Affiliations:** Institute of Oceanography, University of Gdańsk, Gdańsk, Poland

**Keywords:** sea ice, granular rheology, sea ice rheology, floe size distribution, polydispersity, marginal ice zone

## Abstract

Sea ice in the marginal ice zone (MIZ) consists of relatively small floes with a wide size span. In response to oceanic and atmospheric forcing, it behaves as an approximately two-dimensional, highly polydisperse granular material. The established viscous-plastic rheologies used in continuum sea ice models are not suitable for the MIZ; the collisional rheology, in which sea ice is treated as a granular gas, captures only one aspect of the granular behaviour, typical for a narrow range of conditions when dynamics is dominated by binary floe collisions. This paper reviews rheology models and concepts from research on granular materials relevant for MIZ dynamics (average stress as a result of ‘microscopic’ interactions of grains; μ(I) and collisional rheologies). Idealized discrete-element simulations are used to illustrate granular effects and strong influence of the floe size distribution on strain–stress relationships in sheared sea ice, demonstrating the need for an MIZ rheology model capturing the whole range of ‘regimes’, from quasi-static/dense flow in the inner MIZ to the inertial flow in the outer MIZ.

This article is part of the theme issue ‘Theory, modelling and observations of marginal ice zone dynamics: multidisciplinary perspectives and outlooks’.

## 1. Introduction

Sea ice in the marginal ice zone (MIZ)—the part of the ice cover adjacent to the open ocean and influenced by its presence—typically consists of relatively small, highly mobile ice floes. Ice fragmentation is a result of many dynamic processes, including breaking by waves, shear deformation and floe collisions, combined with thermodynamic influences, e.g. upwelling of relatively warm water masses at the ice edge. The resulting floe size distributions (FSDs) in the MIZ often are scale-invariant in the size range of relatively small floes and have exponential tails related to the existence of the upper size limit, i.e. they are well described by a tapered power law [1]. The relationship between floe size and dynamic/thermodynamic processes in the MIZ is very complex and mutual: the response of the ice to oceanic and atmospheric forcing is FSD dependent [2,3]. Granular effects in sea ice dynamics have received much attention of researchers in recent years, with several studies exploring the influence of FSDs on selected processes (e.g. eddy formation in the ocean mixed layer and ice melting rates [4]; formation of floe clusters [5,6]; size-dependent response of floes to wind/currents [7]; jamming phenomena [8]; wave energy attenuation [9,10]; and many more), or on large-scale, seasonal and long-term evolution of sea ice in polar regions (e.g. [11–15]). In spite of a substantial progress, however, many aspects of those interactions remain poorly understood [16]. As far as sea ice rheology—the main subject of this paper—is concerned, the attempts to parametrize granular effects in continuum models have been largely limited to the collisional regime, i.e. the relatively narrow range of ice concentrations where, on the one hand, the floes can move freely in open-water spaces between them, but, on the other hand, they collide with their neighbours frequently and energetically enough to produce substantial collisional stress in the ice. Not surprisingly, when the collisional rheology [17–19] is implemented in large-scale sea ice models together with the classic Hibler’s viscous-plastic rheology [20], its effects are noticeable in terms of, e.g. MIZ width and compactness [14], but overall tend to be rather minor. Other continuum rheological models that aim at taking into account the discrete nature of sea ice are suitable for the dense ice pack rather than the MIZ and reproduce selected, isolated aspects of granular behaviour, e.g.dilatancy effects [21], or anisotropy [22]. At smaller scales, smoothed particle hydrodynamics and discrete-element models (DEMs) have been used to assess the suitability of the viscous-plastic rheology and Mohr–Coulomb type yield conditions to granular sea ice flow in various geometries [23–26].

In parallel with significant progress in research on sea ice dynamics at the floe scale, briefly sketched above, substantial developments have taken place in research on granular rheology, including that of dry granular materials and suspensions. One of the main goals of this study is to provide a concise—and, by necessity, rather selective—review of ideas that seem relevant for sea ice and that might inspire further research on MIZ dynamics and, in a further perspective, lead to the development of a MIZ rheology model capturing the whole range of behaviours, from quasi-static, dense flow in the inner MIZ to dilute, inertial flow close to the ice edge. To this end, after introducing basic definitions related to the rheology of viscous fluids in §2a, the assumptions and properties of several variants of the so-called μ(I) rheology are discussed in §2b, followed by the presentation of the formal definition of stress tensor in granular materials in §2c and details of the already mentioned collisional sea ice rheology (§3). One of the very important aspects of those subjects are effects of polydispersity (i.e. heterogeneity of grain sizes) and methods to account for them in models. Most granular rheologies are formulated for materials with a narrow grain size distribution and their applicability to strongly polydisperse materials remains unexplored. Therefore, insights obtained for sea ice can serve a wider purpose as a test case for granular materials in general. The idealized DEM simulations of simple-shear deformation presented in §4 illustrate some effects not captured by the present sea ice rheology models. In particular, it is shown that the apparent friction coefficient is strongly shear-rate and floe size dependent, and the influence of the largest floes (i.e. the tail of the FSD) on the yield stress, strain–stress relationship and velocity fluctuations is investigated. In §5, conclusions are formulated and possible further directions of research discussed.

## 2. Selected aspects of rheology of dense granular materials

Anticipating the application of concepts presented in this section to continuum modelling of sea ice, in which the momentum and constitutive equations are formulated in two dimensions (integrated over ice thickness), the description and formulae in §2a–c below are given for two-dimensional materials as well. Full three-dimensional versions can be found in the cited papers. Cartesian coordinates are used in the horizontal plane, with axes $x_{i}$, i=1,2.

### (a) Basic definitions: stress and strain in viscous fluids

Continuum models of granular materials, analogously to models of viscous fluids, are based on the conservation equations for mass and momentum, the general form of which is

2.1 $$\frac{D\rho_{b}}{Dt}+\rho_{b}\left(\frac{\partial u_{1}}{\partial x_{1}}+\frac{\partial u_{2}}{\partial x_{2}}\right)=0\;\text{and}\;\frac{D(\rho_{b}u_{i})}{Dt}=\frac{\partial \sigma_{i1}^{c}}{\partial x_{1}}+\frac{\partial \sigma_{i2}^{c}}{\partial x_{2}}+\rho_{b}F_{i}\;\text{for}\;i=1,2,$$

where for any function $f(x_{1},x_{2},t)$ the material derivative $Df/Dt=\partial f/\partial t+u_{1}\partial f/\partial x_{1}+u_{2}\partial f/\partial x_{2}$, t denotes time, $u_{i}$ denotes the velocity component in the direction $x_{i}$, $\rho_{b}$ is the bulk mass density ($\rho_{b}=\rho \phi$, with ρ the mass density of the grains and ϕ the solid volume fraction), $F_{i}$ is the ith component of an external body force (per unit mass), and $\sigma_{ij}^{c}$ are components of the Cauchy stress tensor, resulting from external contact forces acting on the surface of the analysed volume (see, e.g., [27]). Apart from equations (2.1), an essential component of continuum models of granular materials are constitutive equations relating stress to deformation rates and bulk material properties. The rate of deformation tensor ${\dot{\varepsilon }}_{ij}$ is defined in terms of the velocity gradients as

2.2 $${\dot{\varepsilon }}_{ij}=\frac{1}{2}\left(\frac{\partial u_{i}}{\partial x_{j}}+\frac{\partial u_{j}}{\partial x_{i}}\right),$$

where $u_{i}$ denotes the velocity component in the direction $x_{i}$. For the purpose of the further analysis, it is convenient to introduce the following notation: ${\dot{\varepsilon }}_{i}\equiv {\dot{\varepsilon }}_{ii}=\partial u_{i}/\partial x_{i}$ for i=1,2, and $\dot{\gamma }\equiv {\dot{\varepsilon }}_{12}={\dot{\varepsilon }}_{21}=\frac{1}{2}(\partial u_{1}/\partial x_{2}+\partial u_{2}/\partial x_{1})$. Thus, ${\dot{\varepsilon }}_{i}$ and $\dot{\gamma }$ represent the longitudinal and shear strain rates, respectively. The volumetric strain rate is ${\dot{\varepsilon }}_{V}={\dot{\varepsilon }}_{1}+{\dot{\varepsilon }}_{2}$, and the pure-shear strain rate ${\dot{\varepsilon }}_{\text{PS}}={\dot{\varepsilon }}_{1}-{\dot{\varepsilon }}_{2}$. The Cauchy stress tensor $\sigma_{ij}^{c}$ is split into two parts, the isotropic pressure $p^{c}$ and the deviatoric stress ${\hat{\sigma }}_{ij}^{c}$

2.3 $$\sigma_{ij}^{c}=-p^{c}\delta_{ij}+{\hat{\sigma }}_{ij}^{c},$$

where $\delta_{ij}$ denotes the Kronecker delta and ${\hat{\sigma }}_{11}^{c}+{\hat{\sigma }}_{22}^{c}=0$. Analogously to ${\dot{\varepsilon }}_{i}$ and $\dot{\gamma }$, the normal and tangential stress components are denoted with ${\hat{\sigma }}_{i}^{c}\equiv {\hat{\sigma }}_{ii}^{c}$ (for i=1,2) and $\tau^{c}\equiv {\hat{\sigma }}_{12}^{c}={\hat{\sigma }}_{21}^{c}$, respectively. The friction coefficient is $\mu =\tau^{c}/p^{c}$. For compressible viscous fluids [27]

2.4 $${\hat{\sigma }}_{i}^{c}=2\eta {\dot{\varepsilon }}_{i}+(\zeta -\eta ){\dot{\varepsilon }}_{V}\;\text{and}\;\tau^{c}=2\eta \dot{\gamma },$$

where η is the shear (or dynamic) viscosity and ζ is the bulk viscosity, representing the resistance of a fluid towards rapid changes of volume. In Newtonian, i.e. linearly viscous fluids, η is a scalar. In non-Newtonian fluids, η represents the apparent viscosity and is a function of $\dot{\gamma }$, material properties and, in time-dependent rheologies, time. Time-independent fluids can be broadly divided into purely viscous fluids, for which $\tau^{c}(\dot{\gamma }=0)=0$, and viscoplastic fluids, exhibiting a yield shear stress, i.e. $\tau^{c}(\dot{\gamma }=0)>0$. Notably, the Hibler’s viscous-plastic sea ice rheology [20,28] and the collisional rheology described further in §3 both have the general form of (2.3) and (2.4).

### (b) μ(I) rheology

Based on a dimensional analysis of the governing equations describing a simple-shear flow (${\dot{\varepsilon }}_{\text{PS}}={\dot{\varepsilon }}_{V}=0$) of dry granular materials, it has been shown that the friction coefficient μ and the solid volume fraction ϕ are functions of a single, non-dimensional parameter I called the inertial number (e.g. [29])

2.5 $$I=\sqrt{\frac{\rho }{p^{c}}}\dot{\gamma }\bar{d},$$

where $\bar{d}$ is a measure of the average grain diameter (discussed below). Thus, μ=μ(I) and ϕ=ϕ(I) [29–32]. The form of these functions depends on the material properties and, when this rheology model is extended to more complex types of flows, the flow geometry. In three dimensions, $\sigma_{ij}^{c}$ is given by (2.3) with ${\hat{\sigma }}_{ij}^{c}$ [30]

2.6 $${\hat{\sigma }}_{ij}^{c}=\mu (I)p^{c}\frac{{\dot{\varepsilon }}_{ij}}{|\dot{\varepsilon }|},$$

where $|\dot{\varepsilon }|$ is the second invariant of ${\dot{\varepsilon }}_{ij}$. Hence, the apparent viscosity η depends on both shear rate and pressure. In general, suitable candidates for μ(I), ϕ(I) should reflect the two closely related characteristic properties of dense granular flows: shear-thickening and dilatancy. In other words, they should reflect an increase of the apparent viscosity and volume with increasing shear rates, as the flow evolves from the quasi-static through the dense to the kinetic regime. Widely used phenomenological formulae for μ(I) and ϕ(I) are (e.g. [31])

2.7 $$\mu (I)=\mu_{0}+\frac{\left(\mu_{\infty }-\mu_{0}\right)}{\left(I_{0}/I+1\right)}\;\mathrm{and}\;\phi (I)=\phi_{0}-c_{\phi }I,$$

where $\mu_{0}$, $\mu_{\infty }$, $I_{0}$, $\phi_{0}$, $c_{\phi }$ are adjustable coefficients. Thus, ϕ decreases linearly with I from its maximum value $\phi_{0}$, and μ varies between $\mu_{0}$ in the quasi-static regime (I→0) and $\mu_{\infty }$ in the kinetic regime (I→∞). Both $\mu_{0}$ and $\mu_{\infty }$ are finite, the behaviour is viscoelastic, i.e. $\tau (\dot{\gamma }=0)=\mu_{0}p^{c}$. In spite of several limitations (non-locality, long-range correlations, hysteresis effects, etc. typical of granular materials, are not taken into account), as well as problems with applicability of that model to highly inhomogeneous and/or non-stationary flows, and with ill-posedness under some strain combinations [33], the μ(I) rheology has been found in several studies to satisfactorily represent observational and model data (e.g. [31,34,35]) over a wide range of confining pressures, inertial numbers and materials used, although the spread of values of the fitted coefficients obtained in those studies tends to be rather large and the form of μ(I) is in some materials more complex than in (2.7), see, e.g., [36,37] for cohesive media. Overall, the μ(I) rheology performs particularly well for dense flows at low inertial numbers.

From the point of view of the potential application of a μ(I)-type rheology to sea ice (and to highly polydisperse materials in general), an important aspect of the definition of I is its dependence on $\bar{d}$. In the theoretical, numerical and observational studies that led to the formulation of the μ(I) rheology, narrow grain size distributions were considered, with the average grain diameter used for $\bar{d}$, and little attention was paid to the possible effects of polydispersity. More recently, e.g. in studies on rheology of bidisperse mixtures, $\bar{d}$ is usually computed as a volume-fraction-weighted average grain diameter, although little is known about the suitability of the μ(I) rheology and the meaning of $\bar{d}$ in materials with very strong polydispersity (see, e.g. discussion in [38]).

It is also worth noting that if ϕ(I) is invertible, as in (2.7), I can be computed from ϕ so that, combined with (2.5), the pressure $p^{c}$ is given as $p^{c}=p^{c}(\phi ,\dot{\gamma })$, i.e. it depends on both the volume fraction and strain rate (as long as ϕ is sufficiently far from $\phi_{0}$ and $\dot{\gamma }\neq 0$). The relationship $p^{c}(\phi ,\dot{\gamma })$, together with (2.6), makes the rheology applicable in continuum models, where $p^{c}$ and ${\hat{\sigma }}_{ij}^{c}$ have to be computed from available grid-scale variables.

Finally, the inertial number squared, $I^{2}=\rho {\dot{\gamma }}^{2}{\bar{d}}^{2}/p^{c}$, is a ratio of the bulk measure of the inertial pressure to the confining pressure in the material. Based on the additive property of stress, it has been shown recently that the μ(I) rheology can be extended to a wide range of cohesive materials and suspensions (i.e. submerged flows), provided that I is computed in a more general way, i.e. as a ratio of the linear combination of all stresses related to the grains’ motion to the linear combination of stresses related to grain contacts [39,40]. Thus—again, importantly for potential applications to sea ice—cohesive and viscous interactions associated with the presence of fluid in spaces between grains can be included together with the ‘standard’ elastic and frictional grain–grain interactions.

### (c) Definition of stress tensor in granular materials

DEM methods are an invaluable source of information on granular flows as they allow to relate processes acting at different scales, from grain-level interactions, through contact/force chains and grain clusters, to bulk material properties relevant for continuum models. Obtaining that multi-scale information from observational data is an unattainable goal. Obviously, in order for DEM results to be useful, the relevant bulk variables must be computed from information available at the grain level in a proper way. Considering the wide popularity of DEM methods, it might seem surprising that misconceptions regarding the computation of volume-averaged stress are quite common, and several papers are devoted to alternative definitions of that quantity and their interpretation. It is therefore useful to provide here a summary of that computation and the relevant concepts.

In the momentum equations (2.1), there are two terms that are directly related to processes acting at the grain level. One is the divergence of the Cauchy strain tensor on the right-hand side of (2.1): as forces in granular materials are transmitted through grain–grain interactions, the stress applied to the surface of a given volume of that material can be expressed as a sum of grain–grain stresses within that volume, so that, for an assembly of grains occupying a given volume V the Cauchy (or contact) stress is (e.g. [41,42])

**Figure 1.**
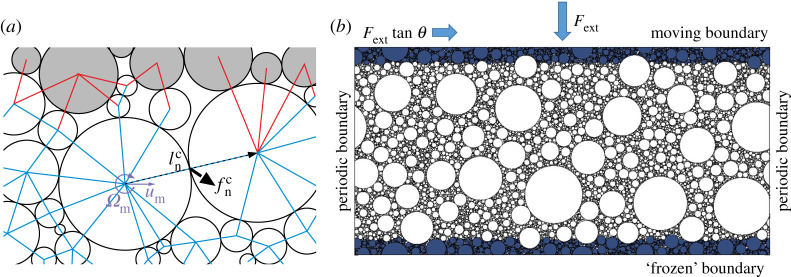
Stress in a sheared granular material. (*a*) the averaged stress as a sum of two components: contact forces between grains within the control volume V (blue lines, white circles) and with boundary grains (red lines, grey circles); and kinetic energy of grains in V (violet arrows). (*b*) a standard DEM set-up for studying strain–stress relationships under simple-shear deformation (see §4a). (Online version in colour.)

2.8 $$\sigma_{ij}^{c}=\frac{1}{V}\sum_{n=1}^{N_{c}}f_{n,i}^{c}l_{n,j}^{c},$$

where $N_{c}$ is the total number of contacts (within V, as well as contacts with ‘outside’ grains through the boundary of V; see figure 1*a*), $f_{n,i}^{c}$ denotes the ith component of the contact force at the nth contact, and $l_{n,j}^{c}$ is the jth component of the vector connecting the centres of mass of grains participating in that contact. The second of the two terms mentioned above is the advective term in the material derivative on the left-hand side of (2.1), which after some rearrangements can be expressed as $\rho_{b}(\partial (u_{i}u_{1})/\partial x_{1}+\partial (u_{i}u_{2})/\partial x_{2})$. In many applications, it is useful to split the instantaneous velocity $u_{i}$ into its mean ${\bar{u}}_{i}$ and fluctuating part $u_{i}^{\prime }$, related to the motion of individual grains: $u_{i}={\bar{u}}_{i}+u_{i}^{\prime }$. By definition, $\bar{u_{i}^{\prime }}=0$, so that the advective term becomes $\rho_{b}(\partial \bar{u_{i}u_{1}}/\partial x_{1}+\partial \bar{u_{i}u_{2}}/\partial x_{2})+\rho_{b}(\partial \bar{u_{i}^{\prime }u_{1}^{\prime }}/\partial x_{1}+\partial \bar{u_{i}^{\prime }u_{2}^{\prime }}/\partial x_{2})$. In a continuum model, the first part of this sum can be directly computed from variables resolved at the grid-level. The second part, analogous to the Reynolds stress in models of turbulent flows, describes the momentum flux due to ‘random’, sub-grid-scale grain motion. As it has an analogous form to the Cauchy stress term, they can be combined into one tensor $\sigma_{ij}^{t}=\sigma_{ij}^{c}+\sigma_{ij}^{k}$, where $\sigma_{ij}^{k}=\rho \phi \bar{u_{i}^{\prime }u_{j}^{\prime }}$ is the momentum flux (or kinetic stress) tensor. For finite-size grains, $\bar{u_{i}^{\prime }u_{j}^{\prime }}$ contains contributions from the linear and angular velocities of the grains (see [43] and [41] for detailed derivation), so that

2.9 $$\sigma_{ij}^{k}=-\frac{1}{V}\sum_{n=1}^{N_{g}}m_{n}u_{n,i}^{\prime }u_{n,j}^{\prime }+\frac{1}{2V}\sum_{n=1}^{N_{g}}M_{n}\Omega_{n}^{2}\delta_{ij},$$

where $N_{g}$ is the number of grains, $m_{n}$ and $M_{n}$ denote the mass and moment of inertia of the nth grain, respectively (for a disc $M_{n}=\frac{1}{2}m_{n}r_{n}^{2}$, where $r_{n}$ is the disc’s radius), and $\Omega_{n}=|\Omega \;\Omega_{n}|$. The rotational component of $\sigma_{ij}^{k}$, equal to the average spin kinetic energy, can be interpreted as a tensile stress related to the centrifugal effects of grains’ rotation (note that this term is always positive, i.e. it has the opposite sign to the contact pressure; its contribution to the shear stress is zero).

Both (2.8) and (2.9) can be directly computed from the results of DEM models and belong to the standard output of most available DEM codes. The already mentioned controversies are related not to the computation of $\sigma_{ij}^{c}$, $\sigma_{ij}^{k}$ and $\sigma_{ij}^{t}$, but rather to their usage and interpretation. In particular, the first part of the kinetic stress in (2.9) is present only if stress is formulated in the Eulerian frame of reference and vanishes if a Lagrangian approach is used (compare the derivation and conclusions in [41,43]). Similarly, $\sigma_{ij}^{c}$ alone should be used when the quantity of interest is the stress exerted by a granular material on walls and other structures, but both $\sigma_{ij}^{c}$ and $\sigma_{ij}^{k}$ should be parametrized in continuum models based on slowly varying variables. As far as $\sigma_{ij}^{k}$ is concerned, computation of ${\bar{u}}_{i}$ and $u_{i}^{\prime }$ is non-trivial if the flow is very complex, non-stationary and, especially, if the sizes of grains are not very small compared with the size of the domain (as will be the case in simulations described in §4). Therefore, in some studies $\sigma_{ij}^{k}$ is computed from the total velocities $u_{i}$ instead of $u_{i}^{\prime }$. This amounts to including the whole advective term in the stress-divergence term on the right-hand side of (2.1); accordingly, it should be removed from the left-hand side. Moreover, although, as is often noted (e.g. [43]), any non-divergent component can be added to $\sigma_{ij}^{k}$ without any influence on stress divergence occurring in the momentum equation, that operation obviously has a substantial influence on the stress itself. In particular, it makes any inferences on the relative contribution of kinetic and contact stresses to the total stress meaningless. While in many cases $\sigma_{ij}^{k}\ll \sigma_{ij}^{c}$ when $\sigma_{ij}^{k}$ is computed based on velocity anomalies, it is not true when the mean advection is included in the momentum-flux tensor. In general, even if $u_{i}^{\prime }$ are used, the ratio $\sigma_{ij}^{k}/\sigma_{ij}^{c}$ is very sensitive to the computation of ${\bar{u}}_{i}$ and $u_{i}^{\prime }$. From the point of view of the momentum balance, the divergence of $\sigma_{ij}^{c}$ and $\sigma_{ij}^{k}$ can be compared, not the stresses themselves. Similar caution should be taken when computing the friction coefficient μ. It was defined in §2a as $\mu =\tau^{c}/p^{c}$. An analogous version $\mu^{t}=(\tau^{c}+\tau^{k})/(p^{c}+p^{k})$ suffers from the problems described above and its usefulness is therefore rather limited.

## 3. Collisional rheology for sea ice

The collisional rheology has been formulated in the 1980s in a series of papers by Shen and colleagues [17,18,44] and subsequently extended by [45,46] and others. The original model was formulated for sea ice with ice concentration A, composed of identical disc-shaped floes with diameters d, thickness h, mass density ρ and restitution coefficient ϵ, and subject to deformation described by a strain rate tensor ${\dot{\varepsilon }}_{ij}$, defined in (2.2). Based on equations describing the momentum transfer and energy dissipation during inelastic floe–floe collisions, with several assumptions regarding the geometry of the problem (see further), [18] obtained the formula for the collisional stress tensor $\sigma_{ij}^{C}$ in the form (2.3) and (2.4), with p, η and ζ given by

3.1 $$p=\frac{\psi }{2^{1/2}\pi }{\left(\frac{v^{\prime }}{\bar{d}}\right)}^{2},\;\eta =\frac{\psi }{3}\frac{v^{\prime }}{\bar{d}},\;\zeta =3\eta ,\;\text{with}\;\psi =\frac{\rho {\bar{d}}^{2}h(1+\epsilon )}{4\pi }f(A),$$

where f(A) is a function of the actual and maximum possible ice concentration $A_{0}$ (equal to 0.91 for equal-size discs), $f(A)=A^{3/2}/(A_{0}^{1/2}-A^{1/2})$, $v^{\prime }$ denotes the ensemble average magnitude of the fluctuation velocity of the discs, and $\bar{d}=d$ (for a more general formulation valid for polydisperse sea ice with relatively narrow FSDs, see [45]). A fundamental assumption underlying the above model is that sea ice behaves as a granular gas in which ‘cooling’ due to inelastic collisions is balanced by ‘heating’ by shear, and the floes do not change their velocities between collisions. Additional assumptions are, first, a predetermined form of the directional distribution function of floes’ fluctuations, and second, large magnitude of those fluctuations compared with the mean shear rate, or to differences between the mean velocities of interacting floes: $v^{\prime }\gg \dot{\gamma }\bar{d}$. This allows to parametrize the ratio $v^{\prime }/\bar{d}$ in (3.1) as a function of ϵ and ${\dot{\varepsilon }}_{ij}$. However, although this condition is typically fulfilled in the MIZ [18,45], the model suffers from limitations that are intrinsic features of granular gases, including the inelastic collapse, i.e. infinitely high collision rates as $A\to A_{0}$ (although, at the same time, high compactness is required for the estimation of collision rates to be valid). A related problem is the assumption of the uniform distribution of floes on the sea surface, which is realistic only if ϵ is sufficiently high to prevent clustering instability (e.g. [47]). Whereas high ϵ values were used in [18,19], more recent field and laboratory studies suggest that collisions in real sea ice are close to inelastic ([48,49] and references there).

A substantial development of the collisional rheology was relaxing the simple-shear assumption of [18]. Based on suggestions formulated in the original paper [18] and research on flows of dense granular materials [50,51], Feltham [19] developed a sea ice model for the MIZ with a combined plastic (Hibler’s) and collisional rheology in which the simplified estimation of $v^{\prime }$ was replaced with a full evolution equation for the granular temperature $T\equiv v^{\prime 2}/2$. The equation includes advection and diffusion terms, as well as source functions describing the input of T due to the work done by the forces from the ocean and atmosphere, and dissipation due to floe–floe interactions (see [19] for details and derivation). However, the computation of those source terms requires several assumptions, some of which are grounded in research on dry granular materials that is not necessarily directly transferable to sea ice, and others, rather debatable.

## 4. Role of floe size distributions in shear deformation in marginal ice zone

### (a) Model set-up

As mentioned in the introduction, a DEM model is used in this study to illustrate selected aspects of the discussed processes. To this end, the DESIgn model [52] is run in an idealized configuration suitable for an analysis of simple-shear scenarios, routinely used for studying rheological properties of granular materials ([29,36,37,39,53,54] and many more). For each group of model runs, a set of $N_{g}$ disc-shaped grains (ice floes) with the tapered power law FSD p(r) is generated with the transformation method described in [55]

4.1 $$p(r)∼r^{-\alpha }\exp\left(\frac{-r}{\beta }\right),$$

where the exponent α and the scale parameter β are both positive. The total surface area $S_{\text{tot}}$ of each floe set is constant, $S_{\text{tot}}=1500^{2}\;m^{2}$, but the sets differ in terms of α and β (and thus the value of $N_{g}$). Hence, they can be interpreted as the same area of ice broken into floes of different sizes. For the combinations of α and β considered (1.0, 1.2, …, 2.0 and 20, 30, 50, 75, 100, 150, 200 m, respectively), the minimum floe diameter $d_{\text{min}}$ remains fairly constant at approximately 2 m, but the maximum diameter $d_{\text{max}}$ varies roughly linearly with β (and independently of α) from approximately 220 m for β=20 m to ∼870 m for β=200 m. Hence, the surface area of the largest floe varies from ∼1.5% to over 26% of $S_{\text{tot}}$. For all sets, the so-called size span $s=(1-d_{\text{min}}/d_{\text{max}})/(1+d_{\text{min}}/d_{\text{max}})$, often used as a measure of polydispersity of granular materials, exceeds 0.98, and is thus larger than in most studies where the influence of s on the bulk material properties is analysed (see [56,57] and references there). The surface-area-weighted floe diameter ${\bar{d}}_{a}$ is

4.2 $${\bar{d}}_{a}=\frac{2\int_{0}^{\infty }r^{3}p(r)\;dr}{\int_{0}^{\infty }r^{2}p(r)\;dr}=2\beta (3-\alpha ).$$

All floes have the mass density $\rho =920\;\mathrm{kg}\;m^{-3}$, thickness h=0.5 m, elastic modulus $E=5\times 10^{9}\;\mathrm{Pa}$, Poisson’s ratio ν=0.33 and friction coefficient $\mu_{g}=0.7$. All details of the contact model and its numerical implementation can be found in electronic supplementary material of [52].

During the preparation phase of each run group, the floes are placed randomly, without overlaps, within a square domain with surface area $S_{\text{tot}}/0.85$, which is then uniformly compressed to a smaller area $S_{\text{tot}}/0.90$ (so that the domain dimensions are $L_{x_{1}}=L_{x_{2}}=1581\;m$) and left to relax over a period of time sufficient for the stresses and velocities to decrease to insignificantly small values. Subsequently, the domain is divided into three regions (figure 1*b*): the lower, ‘frozen’ boundary for $x_{2}<0.1L_{x_{2}}$, where the velocities of all floes are set to zero; the upper boundary for $x_{2}>0.9L_{x_{2}}$, where the floes move collectively as one rigid body subject to an external force $-F_{\text{ext}}$ in the $x_{2}$-direction and $F_{\text{ext}}\tan\theta$ in the $x_{1}$-direction; and the middle region, where the full linear and angular momentum equations are solved for each floe (see [52]). The boundaries in the $x_{1}$-direction are periodic, $L_{x_{1}}$ is kept constant at the value given above, and $L_{x_{2}}$ varies accordingly to the applied confining pressure. Three values of $F_{\text{ext}}$ are considered, producing the average pressure in the material $p_{1}∼60\;\mathrm{kPa}$, $p_{2}∼6\;\mathrm{kPa}$ and $p_{3}∼0.6\;\mathrm{kPa}$, respectively (which corresponds to the internal ice pressure resulting from a moderate on-ice wind stress of 0.3 Pa acting over the distance of 100 km, 10 km and 1 km; thus $p_{1}$ represents pressure conditions in the inner MIZ, and $p_{3}$ conditions close to the ice edge). For each set of ice floes and each $F_{\text{ext}}$, θ is increased from 0 to $24^{\circ }$ with steps of $1.5^{\circ }$, and the evolution of the global and per-floe variables of interest is recorded for further analysis, described in §4b. In particular, with $\bar{d}={\bar{d}}_{a}$ given by (4.2) and the domain-averaged shear rate $\dot{\gamma }=V_{\text{top}}/L_{x_{2}}$, where $V_{\text{top}}$ is the simulated, time averaged $x_{1}$-velocity of the upper boundary, the inertial number (2.5) becomes

4.3 $$I=\frac{2\beta (3-\alpha )V_{\text{top}}}{L_{x_{2}}}\sqrt{\frac{\rho }{p^{c}}}.$$

The contact pressure $p^{c}$, shear stress $\tau^{c}$ and friction coefficient μ are computed from $\sigma_{ij}^{c}$ defined in (2.8). Notably, under stationary conditions in the simple-shear scenario, the momentum equation (2.1) reduces to the balance between the divergence of the contact and kinetic stresses (with, possibly, the external body force added). Therefore, the analysis in §4b below concentrates on the contact stresses, with references to the velocity anomalies and momentum flux when it adds to the understanding of the underlying dynamics.

All simulations are repeated in two versions: with boundary forcing only (version A); and with ice–water drag, assuming ocean at rest (version B). In version B, the ith component of the drag force on a floe with radius r is $F_{w,i}=-\pi r^{2}\rho_{w}C_{d,w}|u|u_{i}$, $\rho_{w}=1027\;\mathrm{kg}\;m^{-3}$ is water density, $C_{d,w}=5\times 10^{-3}$ is water–ice drag coefficient and |u| is the floe’s speed.

### (b) Results

**Figure 2.**
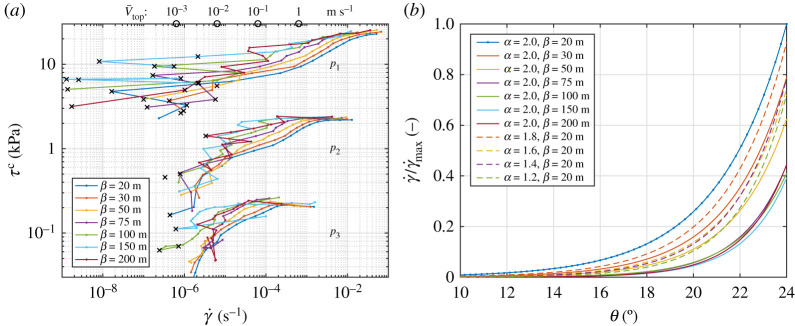
Influence of FSDs on the model response to shear deformation: shear stress $\tau^{c}$ versus shear rate $\dot{\gamma }$ for α=2.0 and the three values of pressure considered *(a*); and the dependence of $\dot{\gamma }$ on FSD properties for selected combinations of α and β (*b*). In (*a*), black crosses mark data points for which variance of $V_{\text{top}}$ exceeds its mean value. For orientation, circles at the upper boundary show approximate values of $V_{\text{top}}$ (computed from average $L_{x_{2}}$ over all runs) corresponding to $\dot{\gamma }$ at the bottom axis.In (*b*), all values are scaled with ${\dot{\gamma }}_{\text{max}}$, the largest strain rate among all cases considered (for α=2.0, β=20 m and $\theta =24^{\circ }$). (Online version in colour.)

The model version A, without any forces from the ocean or atmosphere, represents a dry granular material (and is thus analogous to the treatment of sea ice in the collisional rheology). The results of that version are very useful as a reference case, for which the role of polydispersity in the material’s response to shear deformation can be analysed in detail. Figure 2*a* shows the shear stress $\tau^{c}$ in function of $\dot{\gamma }$ and β for the three confining pressure values considered. Roughly, three regions of $\left(\dot{\gamma },\tau^{c}\right)$ can be distinguished in each group of curves: the quasi-static regime, where $\dot{\gamma }$ is very small and the motion of the upper boundary results from local rearrangements of groups of grains rather than from the net deformation of the sample, so that the variance of $V_{\text{top}}$ exceeds its mean value (black crosses in figure 2*a*; notably, some rearrangements are present even at θ=0, i.e. when no macroscopic deformation is occurring); the dense flow regime, where $\tau^{c}$ increases smoothly with $\dot{\gamma }$ and with β; and the rapid flow regime, in which $\tau^{c}$ saturates towards an approximately constant, floe size- and shear rate-independent value. Characteristically, the range of $\dot{\gamma }$ separating the quasi-static from the rapid flow regime decreases with decreasing confining pressure, and the value of θ for which the material begins to flow increases with increasing β. In other words, the wider the FSD and the higher the $\bar{d}$, the higher the shear pressure the material can sustain without undergoing macroscopic deformation other than very slow creep accommodated by local groups of floes. At intermediate shear rates, for a given value of $\dot{\gamma }$ the shear pressure $\tau^{c}$ differs by a factor of two or more between β=20 m and β=200 m, and vice versa, a given shear stress leads to much slower deformation of ice when the floes are large than when they are small (figure 2*b*).

**Figure 3.**
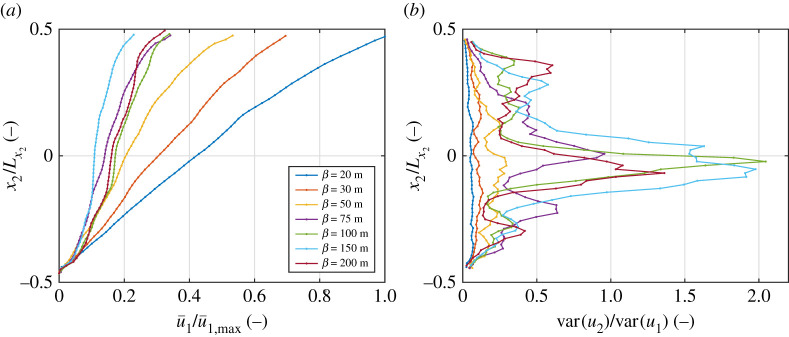
Average velocity profiles ${\bar{u}}_{1}(x_{2}/L_{x_{2}})$ (*a*) and ratio of variances of the two velocity components $\mathrm{var}(u_{2})/\mathrm{var}(u_{1})$ (*b*) for different values of β (colours). In (*a*), all values are scaled with ${\bar{u}}_{1,\text{max}}$, the largest velocity among all cases plotted. Results for α=2.0, $\theta =24^{\circ }$. (Online version in colour.)

Those FSD-related effects, including the decrease of $\dot{\gamma }$ with increasing β and/or decreasing α (figure 2*b*), are closely related to the presence of the largest floes in the ensemble. If all floes are small, $d\ll L_{x_{2}}$, deformation is uniform over the entire domain, the velocity profiles $u_{1}(x_{2})$ are approximately linear and the velocity fluctuations in the $x_{2}$-direction are small compared with those in the $x_{1}$ direction (figure 3*a*,*b*). The presence of floes with diameters comparable with the size of the domain disrupts the uniform character of the flow. As the floes move as rigid bodies, velocity $u_{1}$ in their direct surroundings is $x_{2}$-independent, so that the actual shear deformation has to be accommodated in one or several relatively narrow bands (see the velocity profile for β=200 m in figure 3*a*)—the net flow becomes sensitive not only to the sizes, but also to the relative positions of the small number of the largest floes and, moreover, it has a fully two-dimensional character, i.e. due to small floes flowing around the edges of the large ones, the variance of $u_{2}$ becomes comparable or even larger than the variance of $u_{1}$ (curves for β≥75 m in figure 3*b*). The net, macroscopic result is a larger stiffness of the ice described above, i.e. lower strain rates for a given shear stress or, *vice versa*, higher stress under a given strain rate. Importantly as well, large size span of floes leads to strong temporal fluctuations of stress, related to phases of a relatively smooth flow separated by events of sudden blocking (not shown).

**Figure 4.**
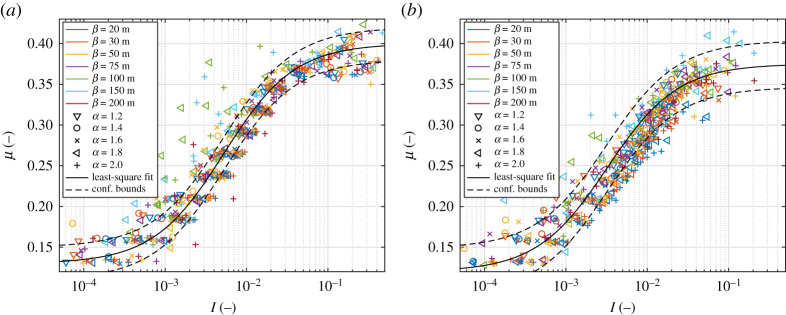
μ(I) for different combinations of α (symbols), β (colours), θ and p in simulations from version A (*a*) and B (*b*).The black curves show the least-square fit of equation (2.7), dashed curves mark the 95% confidence interval. (Online version in colour.)

In spite of the very wide range of I and $\tau^{c}$ obtained for the combinations of FSD properties (α, β) and forcing ($F_{\text{ext}}$, θ) considered (figure 2*a*), the resulting combinations of μ and I all fall onto a relatively well-defined curve (figure 4*a*) which can be well approximated with formula (2.7). Although the fit is not perfect, the majority of the data points lie within the 95% confidence interval. The fitted coefficients for version A are: $\mu_{0}=0.13$, $\mu_{\infty }=0.40$ and $I_{0}=6.8\times 10^{-3}$. Thus, the area-weighted mean floe diameter ${\bar{d}}_{a}$, used in the computation of I, seems a suitable measure of the average floe size. Essentially, all data points in figure 4*a* that strongly deviate from the μ(I) curve correspond to β≥100 m, i.e.situations with the already mentioned localized, transient jamming events that affect the average values of stress and strain, and illustrate the deficiency of the μ(I) rheology in capturing the material behaviour during abrupt changes of strain rates. Overall, however, μ varies smoothly with I and, as will be discussed further in §5, increases from very low values in the quasi-static regime towards much higher values in the inertial regime. The situation does not change substantially when the ice–ocean drag is added (figure 4*b*), although, as expected, the range of obtained shear rates and thus inertial numbers is narrower (the unrealistically high $\dot{\gamma }$ values in version A, figure 2*a*, are removed). A less trivial result is that transient jamming at high ${\bar{d}}_{a}$ is less frequent in version B than in version A (a smaller number of ‘outliers’ in figure 4*b* than in figure 4*a*).

**Figure 5.**
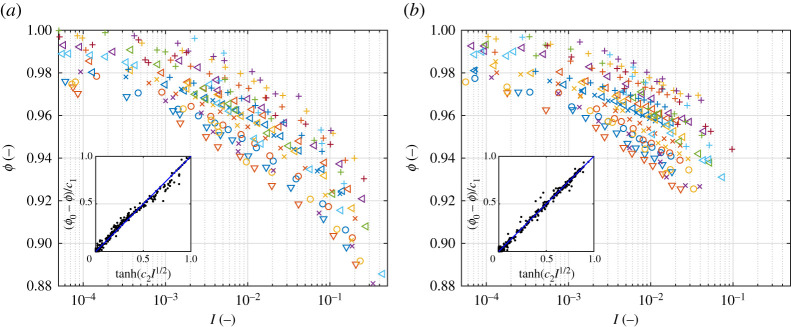
ϕ(I) for different combinations of α, β, θ and p (for legend, see figure 4) in simulations from version A (*a*) and B (*b*). The insets show the least-square fit to the data of function $\phi =\phi_{0}-c_{1}\tanh(c_{2}I^{1/2})$, with $\phi_{0}$, $c_{1}$, $c_{2}$ FSD dependent (see text for details). (Online version in colour.)

Finally, in both versions and in all cases considered the ice concentration A (equivalent to the volume fraction ϕ) decreases with increasing I, but, unlike assumed in (2.7), the relationship is not linear and is FSD dependent (figure 5). As the insets in figure 5 show, $\phi =\phi_{0}-c_{1}\tanh(c_{2}I^{1/2})$ gives a good fit to the data, provided $\phi_{0}$, $c_{1}$, $c_{2}$ are not constant, but functions of α and β. Clearly, although the sensitivity of ϕ to FSD properties is not surprising, this property is disadvantageous in practical applications. On the other hand, however, considering the overall small range of variability of ϕ over a wide range of confining pressures and shear rates, using a single, linear formula (2.7) should be acceptable in practice.

## 5. Discussion and conclusion

Granular materials are mesoscopic—in their dynamics, the microscopic (grain-level) temporal and spatial scales are not well separated from the macroscopic scales. The lack of scale separation is related not only to the fairly large ratio of grain size to the dimensions of systems typically analysed in practical problems (or, equivalently, relatively small number of grains within an analysed volume), but, most importantly, to the nature of interactions between grains [47,58,59]. Thus, mesoscopy is a generic feature of those materials, manifesting itself in phenomena unique to them: a tendency to form clusters of grains, non-local character of macroscopic properties, history effects, scale dependence of stress, long-length correlations associated with force networks, arches of grains, etc. It is not surprising that those effects are amplified in strongly polydisperse materials, in which a wide size span of grains additionally blurs the boundary between micro- and macro-scales: when the grain-size distributions are heavy-tailed, contact and force networks tend to be fractal (see, e.g., [8,54,56,57], and references there). Another feature, closely related to the fractal, ‘openwork’ character of force networks, is the fact that the forces are carried primarily by a small subset of the largest grains. Those grains build a skeleton of the force network and stay in a semi-permanent contact with each other, while smaller grains in regions not participating in force transmission constantly rearrange their positions and take part in relatively short-lived collisions (as analysed in detail in [8]). Therefore, there is no clear boundary between the ‘collisional regime’ and ‘permanent-contact regime’. To the contrary, different types of contacts coexist and the range of applicability of collisional-type rheologies is hard to define.

Not surprisingly, all those properties lead to difficulties in continuum modelling, for which separation of scales is a prerequisite. Yet another difficulty—contrary to those mentioned above related specifically to sea ice—are increasing spatial resolutions of continuum sea ice models, especially those operated at regional and local scales. Although, indisputably, that development is very positive, the drawback is that even in the MIZ, where ice floes are relatively small, continuum rheologies have to deal with floe sizes comparable with mesh sizes of the models. The results presented in this paper suggest that, on the one hand, the average stress can be reliably computed and the strain–stress relationships remain relatively stable up to high floe diameter to mesh size ratios, but, on the other hand, the information on FSD properties is crucial for that computation.

Another information that might play a role, not analysed in this study (and hardly taken into account in sea ice research in general) are floe shapes. In the simulations in §4, the floes were disc-shaped and no rolling resistance was included in the contact model. This might explain the rather low values of friction coefficients obtained (note that no attempt was made to calibrate the model to any real MIZ scenario; a sensitivity analysis to model parameters will be performed in a subsequent study). The sparse evidence available, based on recent ice tank experiments and DEM simulations, indicates that floe shapes influence the stability of force networks and therefore stress in the ice, as well as ice-induced loads on structures (e.g. [60]). At the same time, whereas studies on granular materials with multi-shaped grains generally confirm the sensitivity of force networks and bulk properties to grain shape, they also suggest that shape becomes less important in materials with wide grain size spans (e.g. [61,62]). Applied to sea ice, this indicates that floe shapes might be less important for rheology of ice with wide FSDs—like that analysed in this paper—than that of ice with narrow FSDs, e.g. in ice freshly broken by waves (see [63] for an example).

Apart from the influence of FSDs on stress in the ice, a very robust feature clearly seen in the results presented in this paper (and, again, typical for granular materials) is the dependence of the friction coefficient μ on the shear rate $\dot{\gamma }$. In the Hibler’s sea ice rheology, $\mu =2\dot{\gamma }/(e^{2}\Delta )$, where $\Delta^{2}={\dot{\varepsilon }}_{V}^{2}+e^{-2}({\dot{\varepsilon }}_{\text{PS}}^{2}+4{\dot{\gamma }}^{2})$ and e denotes eccentricity of the elliptical yield curve [20,28]. Thus, in a simple-shear flow $\mu =e^{-1}$ is a constant (equal to 0.5 when a typical value e=2 is used). It is easy to show that in the collisional rheology described in §3 μ is $\dot{\gamma }$-independent as well. In the original formulation [18], $\mu =4\pi /3{\left(v^{\prime }/\bar{d}\right)}^{-1}\dot{\gamma }$, and under simple-shear deformation $v^{\prime }/\bar{d}∼\dot{\gamma }$, which makes μ depend only on the restitution coefficient. In the version of Feltham [19] $v^{\prime }=\sqrt{2T}$ and the granular temperature T is computed from the full turbulent-energy conservation equation. However, when applied to a simple-shear scenario, this model predicts a constant μ as well. Thus, both the Hibler’s and the collisional rheologies disregard the increase of the friction coefficient with shear rate and floe size. Because, as said, this effect is very pronounced in sheared granular flows, a rheology model suitable for the MIZ—as well as for regions in straits, channels or near the coasts, where sheared motion of strongly fragmented ice takes place—should take it into account. In spite of its limitations and deficiencies, a μ(I)-type rheology analysed in this paper seems a good starting point for developing such a rheology model.

## Data Availability

The code of the DESIgn model is freely available on the Internet, see [52]. Input files and configuration scripts necessary to reproduce the results from this paper can be obtained from the author.
